# Parental Education, Household Income, Race, and Children’s Working Memory: Complexity of the Effects

**DOI:** 10.3390/brainsci10120950

**Published:** 2020-12-07

**Authors:** Golnoush Akhlaghipour, Shervin Assari

**Affiliations:** 1 Department of Neurology, University of California Irvine, Irvine, CA 92697, USA; golnoush.akhlaghi@gmail.com; 2 Department of Family Medicine, Charles R Drew University of Medicine and Science, Los Angeles, CA 90059, USA; 3 Department of Urban Public Health, Charles R Drew University of Medicine and Science, Los Angeles, CA 90059, USA

**Keywords:** socioeconomic status, socioeconomic position, memory, working memory, social determinants of health, population groups

## Abstract

***Background.*** Considerable research has linked social determinants of health (SDoHs) such as race, parental education, and household income to school performance, and these effects may be in part due to working memory. However, a growing literature shows that these effects may be complex: while the effects of parental education may be diminished for Blacks than Whites, household income may explain such effects. ***Purpose.*** Considering race as sociological rather than a biological construct (race as a proxy of racism) and built on Minorities’ Diminished Returns (MDRs), this study explored complexities of the effects of SDoHs on children’s working memory. ***Methods.*** We borrowed data from the Adolescent Brain Cognitive Development (ABCD) study. The total sample was 10,418, 9- and 10-year-old children. The independent variables were race, parental education, and household income. The primary outcome was working memory measured by the NIH Toolbox Card Sorting Test. Age, sex, ethnicity, and parental marital status were the covariates. To analyze the data, we used mixed-effect regression models. ***Results.*** High parental education and household income were associated with higher and Black race was associated with lower working memory. The association between high parental education but not household income was less pronounced for Black than White children. This differential effect of parental education on working memory was explained by household income. ***Conclusions.*** For American children, parental education generates unequal working memory, depending on race. This means parental education loses some of its expected effects for Black families. It also suggests that while White children with highly educated parents have the highest working memory, Black children report lower working memory, regardless of their parental education. This inequality is mainly because of differential income in highly educated White and Black families. This finding has significant public policy and economic implications and suggests we need to do far more than equalizing education to eliminate racial inequalities in children’s cognitive outcomes. While there is a need for multilevel policies that reduce the effect of racism and social stratification for middle-class Black families, equalizing income may have more returns than equalizing education.

## 1. Background

Working memory is believed to be a core element of human cognition. Baddeley’s original work on the multiple-component model [1] and executive function conceptualizes working memory as people’s ability to bring information “online”, which is core to thinking and thought. Working memory is our ability to hold information in short-term memory and maintain the required information “in mind” while processing them [2]. Over the past decades, working memory has received much scientific interest, which has resulted in a large body of empirical evidence. This research suggests that working memory is essential for cognitive tasks, math ability, and school performance. Working memory is closely associated with executive functioning [3] and is mainly performed in higher cortical areas, especially the prefrontal cortex (PFC) [4]. Because of the close correlation with cognitive tasks, working memory is believed to be the primary determinant of children’s educational success [5,6,7,8].

With cognitive flexibility and inhibitory control, working memory is a part of the executive function, also called cognitive control [9,10]. Executive function refers to the top-down neurocognitive processes involved in the conscious, goal-directed control of thought, action, and emotions. Effective executive function and functional working memory are both reliant upon the integrity of neural networks involving the prefrontal cortex (PFC), the anterior cingulate cortex, and other regions [11,12,13], and they are required for keeping the information in mind, attending selectively, ignoring distractions, and solving problems flexibly [14].

High socioeconomic status (SES) is associated with better school performance [15] and working memory [16,17,18]. These are in line with the overall positive effects of high SES on childhood cognition, emotions, and behaviors [19]. For example, children from higher SES families are less likely to show school drop-out [20] and emotional [21,22,23] and behavioral problems [24,25]. These SES effects are non-specific and are attributed to the protective effects of resources and lower stress in childhood.

The SES–memory/health scarcity hypothesis can be seen through SES effects on healthy children’s brain development. According to the scarcity hypothesis, low SES is a proxy of early adversity, stress, economic insecurity, and lack of resources, increasing the risk of low child development. In this view, stress, adversity, and scarce resources explain the SES–brain development link [26]. Low parental education and household income are proxies of living in stressful environments, food insecurity, environmental toxins, and parental risk behaviors that can jeopardize healthy brain development in children [27,28,29]. As a result of inadequate brain development, children from low SES are at an increased risk of poor memory, emotion regulation, learning disorders, and psychopathology [30,31,32]. In contrast, children from high SES backgrounds experience less stress and have more access to stimulating environments and better parenting [33,34,35].

According to the Minorities’ Diminished Returns (MDRs) [36,37], Black and White families differ in the protective effect of high SES on health outcomes. Compared to their White counterparts, Black children show lower parental education effects on a wide range of developmental outcomes [38], such as school performance [38], mental health [39], emotion regulation [40,41], aggression [42], and substance use [39,43]. While income may also generate differential effects for Black and White children [44], most research has shown that parental education generates fewer Blacks outcomes than Whites [45,46,47,48,49].

MDRs are not due to behaviors or personalities but societal barriers. For Black families, high SES increases vulnerability to the effect of discrimination, meaning that if discrimination occurs, it is more likely to result in depression [50]. High SES is also associated with high discrimination, which is partly due to the increased proximity of high SES Blacks to Whites [51,52]. High SES Black families experience higher discrimination because they are at higher proximity to White people [51,52,53,54]. The positive link between SES and discrimination [51,52,55,56,57,58] reduces high SES Black families’ health. As a result, SES effects are weaker for Blacks than Whites.

Research has established racial/ethnic differences in each SES indicator’s role in children’s brain development [33,59,60,61]. In several studies, the magnitude of parental education’s effects on a wide range of developmental and health outcomes is weaker for Blacks than Whites [49,55,58,62,63,64,65,66]. As a result of MDRs, middle-class ethnic minority children remain at risk for poor developmental and health outcomes [45,67,68,69,70]. For example, high SES Black children remain at risk of anxiety [71], depression [44], poor health [62], poor school performance [72,73], and high-risk behaviors [45] such as aggression [45] and tobacco use [74,75]. Differential effects of SES across racial and ethnic groups of children are robust [49,62,63,76,77]. Data from the Fragile Families and Child Wellbeing Study (FFCWS) shows that high parental education and family income is associated with better outcomes in impulsivity, school performance, school bonding, attention-deficit/hyperactivity disorder (ADHD), obesity, aggression, depression, and self-rated health for White children than Black American children [69,77,78,79]. Subjective SES and parental education each impact brain imaging findings in a certain way [33,59,60,61]. Various SES indicators may also be the underlying mechanisms by which racial and ethnic disparities emerge in children’s development [49,62,80].

At least some of the effects of high SES on school performance [81] can be attributed to the role of family SES on structure and function of the brain [33] and SES effect on memory [82,83,84]. Many brain structures, such as the amygdala [33,60,85], hippocampus [86], and PFC, carry the effects of SES on cognitive and behavioral outcomes. The amygdala is more involved in emotion regulation [87,88,89,90,91], while the hippocampus [86] and PFC [92,93,94] are more involved in cognitive tasks, executive function, and memory.

### Aims

To investigate the complexities of social determinants of children’s brain development in the US, we explored racial variation in the effects of two family SES indicators, namely parental education and household income, on working memory among 9- and 10-year-old children. We expected racial differences in the magnitude of the association between parental education on working memory, in line with the observed MDRs [36,37,45]. More specifically, we expected the weaker effects of parental education on working memory for Black than White children. This expectation is in line with the other research on a wide range of phenotypes and behaviors [36,37,45].

## 2. Methods

### 2.1. Design and Settings

This secondary analysis was a cross-sectional analysis of the baseline data of the ABCD study [95,96,97,98,99]. ABCD is a national, state-of-the-art brain imaging study of childhood brain development [95,100].

### 2.2. Participants and Sampling

The ABCD study sample was recruited from 21 cities across states. ABCD sampling was primarily through school systems. For sampling in the ABCD study, school selection was informed by race, ethnicity, sex, SES, and urbanicity [101]. Inclusion criteria were having data on our variables. Participants could be included regardless of race or ethnicity (*n* = 10,418). As this is a general population study of children, participants have been enrolled regardless of their psychopathologies. That means participants were not included or excluded from the sample based on the presence of psychopathology.

### 2.3. Study Variables

#### 2.3.1. Primary Outcome

The primary outcome was working memory, measured by NIH Toolbox, the Dimensional Change Card Sort [102]. This measure [103] has shown high reliability and validity [9,104]. The NIH Toolbox card-sorting test is a part of the NIH Toolbox Cognition Battery (NIHTB-CB). This measure evaluates the executive function. The NIHTB-CB is designed for use in epidemiologic studies and clinical trials for ages 3 to 85. Some studies have documented very acceptable psychometric properties of the NIHTB-CB and card sort test. These are computer-based instruments assessing executive function: the Dimensional Change Card Sort, which measures cognitive flexibility, and a flanker task, which measures inhibitory control and selective attention. These measures show convergent and discriminant validity and correlate with SES. These measures also show excellent sensitivity to age-related changes during adulthood, excellent test-retest reliability. As a result, the Dimensional Change Card Sort can be used effectively in epidemiologic and clinical studies. Our outcome was a continuous variable in this study, with a higher score indicating higher cognitive flexibility [103,105].

#### 2.3.2. Independent Variable

*Parental Educational Attainment.* Participants reported their years of schooling. This variable was operationalized as a five-level nominal variable: less than a high school diploma, high school diploma, some college, bachelor degree, graduate studies.

*Household Income.* Parents reported their overall annual income. This was a three-level nominal variable: <$50,000, $50,000–$100,000, and $100,000+.

#### 2.3.3. Moderator

*Race.* Race was reported by parents, and operationalized as a nominal variable: Black, Asian, Other/Mixed, and White (reference group).

#### 2.3.4. Confounders

*Ethnicity.* Parents were asked if they were of Latino ethnic background. This variable was coded as Latino = 1 and non-Latino = 0.

*Age.* Age was a dichotomous variable coded 1 or 0 for 10 years and 9 years of age. Parents reported the age of the children.

*Sex.* Sex was 1 for males and 0 for females.

*Parental marital status.* Parental marital status was 1 for married and 0 for any other condition (reference).

### 2.4. Data Analysis

**Figure 1:**
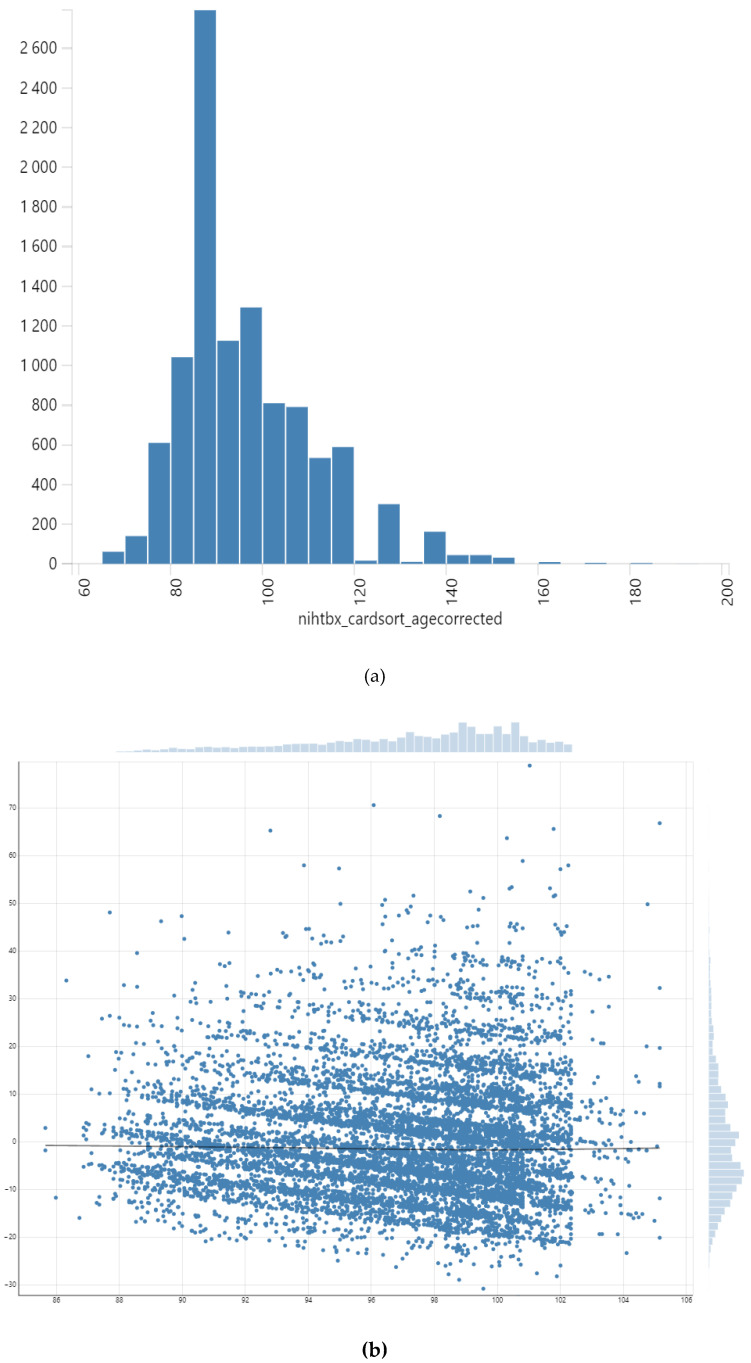

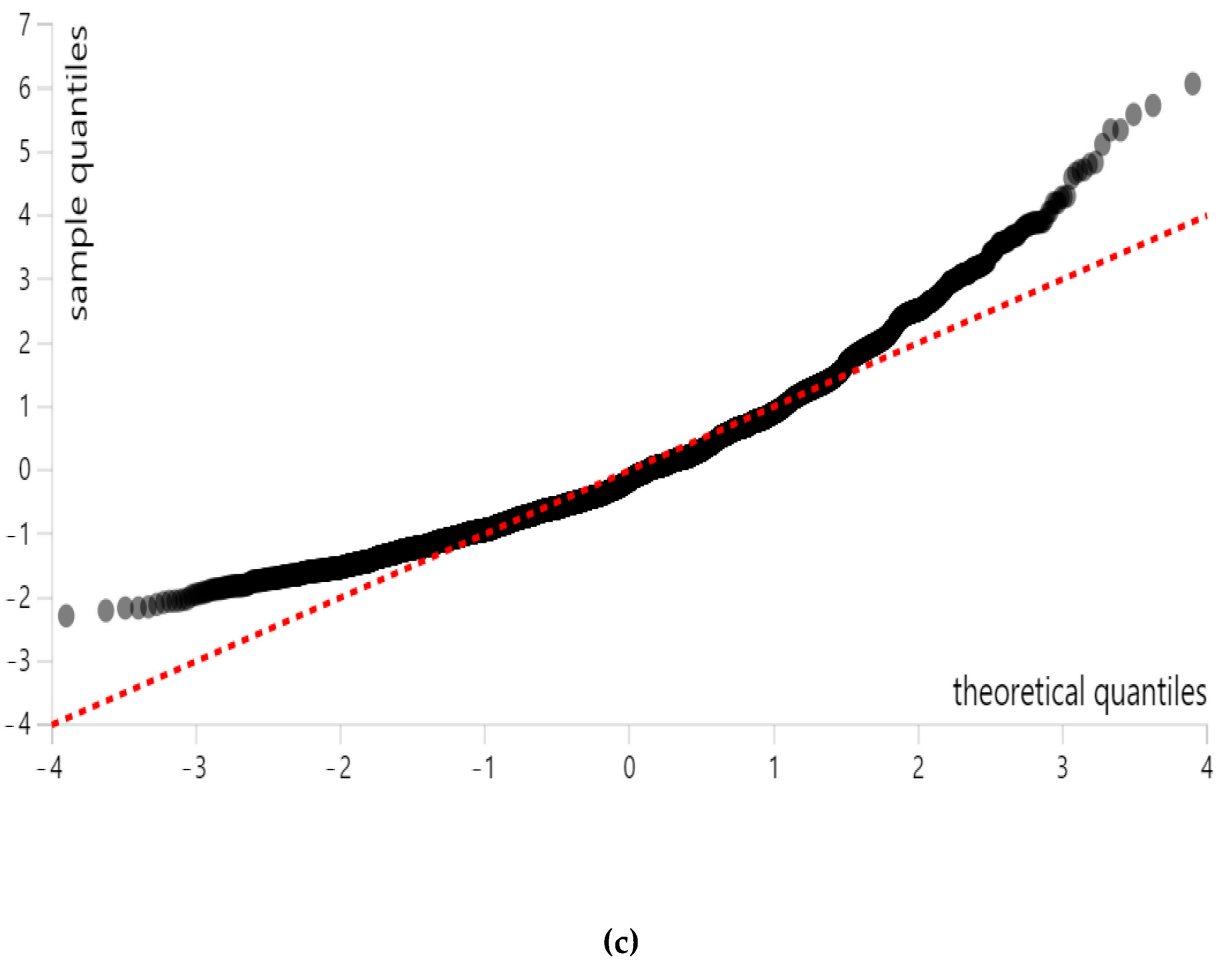
Testing our model’s assumptions: (**a**) distribution of our outcome, (**b**) residuals, and (**c**) quantiles.

We used SPSS for data analysis. Frequencies (n and %) and mean [standard deviations (SDs)] were reported for descriptive purposes. To estimate bivariate associations between the study variables, we used the Chi-square and Analysis of Variance (ANOVA) test in the pooled sample. To perform our multivariable analyses, we performed mixed-effects regressions. First, we tested the assumptions. We excluded collinearity between the study variables. We also tested the distribution of our outcome and error terms and quantiles (Figure 1). We ran six models. All models were performed in the pooled sample. *Model 1* to *Model 3* did not have interaction effects. *Model 1* had education but not income. *Model 2* had income but not education. *Model 3* had both education and income. *Model 4* had interactive effects of education and race but not income. *Model 5* had interactive effects of income and race. *Model 6* had interactive effects of race and education and also controlled for income. Box 1 lists our model formulas. Unstandardized regression coefficient (b), standard error (SE), and *p*-values were reported for each model. A *p*-value of equal or less than 0.05 was significant.

Box 1 **Model Formula.**

**Model 1**

nihtbx_cardsort_agecorrected ~ race.4level + sex + age + married.bl + hisp + high.educ.bl

**Model 2**

nihtbx_cardsort_agecorrected ~ + race.4level + sex + age + married.bl + hisp + household.income.bl

**Model 3**

nihtbx_cardsort_agecorrected ~ + race.4level + sex + age + married.bl + hisp + high.educ.bl + household.income.bl

**Model 4**

nihtbx_cardsort_agecorrected ~ + race.4level + sex + age + married.bl + hisp + high.educ.bl + high.educ.bl × race.4level

**Model 5**

nihtbx_cardsort_agecorrected ~ + race.4level + sex + age + married.bl + hisp + high.educ.bl + high.educ.bl × race.4level + household.income.bl

**Model 6**

nihtbx_cardsort_agecorrected~ race.4level + sex + age + married.bl + hisp + high.educ.bl + household.income.bl + high.educ.bl × race.4level

Random: ~(1|abcd_site/rel_family_id)

### 2.5. Ethical Aspect

Our analysis was exempt from a full review. However, the ABCD study protocol was approved by the University of California, San Diego (UCSD) Institutional Review Board (IRB) [100].

## 3. Results

### 3.1. Descriptives

The sample included 10,418 9- and 10-year-old children. Our participants were White (*n* = 6897; 66.2%), Black (*n* = 1515; 14.5%), Asian (*n* = 234; 2.2%), or other/mixed race (*n* = 1768; 17.0%). Card sorting was significantly different across racial groups. While Asian and White children had the highest card sorting scores, Black children scored worst in card sorting (Table 1).

### 3.2. Regression Results

**Figure 2:**
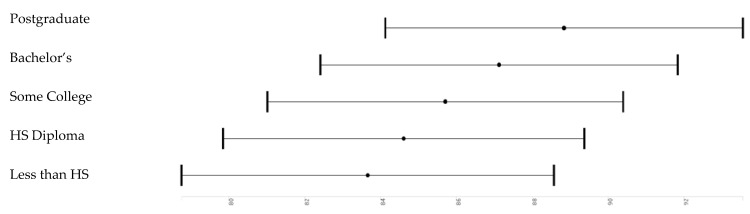
Parental education effects overall.

**Figure 3:**
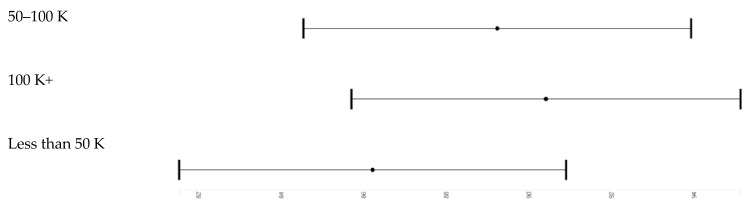
Income effects overall.

**Figure 4:**
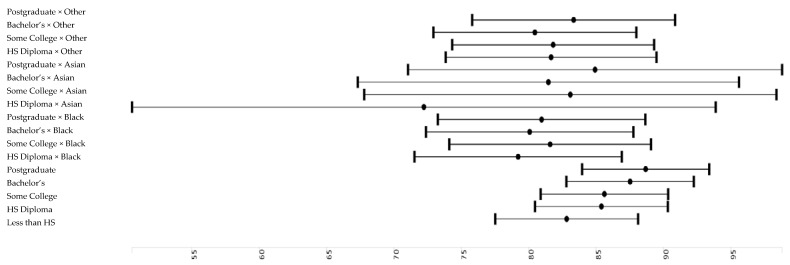
Parental education across groups.

**Figure 5:**
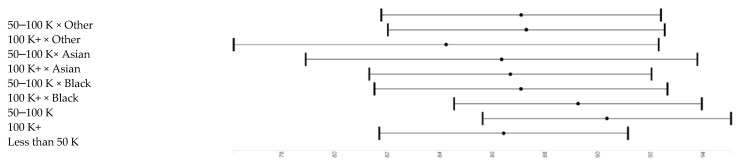
Income effects across groups.

Table 2 and Table 3 report the results of six pooled sample mixed-effects regression models. All models are significant. Effect sizes as shown in Table 2. *Model 1*, which only included the main effect of race and parental education and covariates, showed that high parental education is associated with higher working memory. *Model 2* showed that high income is associated with higher working memory. *Model 3* showed that parental education and household income have both associations with working memory. *Model 4* showed that parental education and race interact, meaning that parental education’s boosting effect on working memory was less pronounced for Black than White children. *Model 5* did not show an interaction between race and household income. *Model 6* showed that household income explains why parental education and race interact with our outcome. (Figure 2, Figure 3, Figure 4 and Figure 5).

## 4. Discussion

This study had three primary findings. Although higher parental education and household income predicted higher working memory (1st finding), parental education and household income showed weaker effects on Black children than White children’s working memory (2nd finding). Third, income differentials (lower income levels of Black families with the same parental education and family structure) explain why parental education shows a weaker effect on working memory for Black than White children (3rd finding).

Our first results can be compared with the literature on the protective effects of high SES on children’s cognitive outcomes [106]. A large body of literature has also documented poor educational outcomes in low SES than high SES children [107,108,109,110]. High SES is associated with better school performance [15] and working memory [16,17,18]. Literature has shown the effects of family SES indicators such as poverty and household income on the brain [111] and behavior [33,59,60,61,112]. The SES–health link may be because SES is a proxy of stress, adversities, trauma. Thus, many brain structures such as PFC, hippocampus, and amygdala [85,113,114,115,116,117,118,119,120,121,122] correlate with SES. The prefrontal cortex [33], hippocampus [86], and amygdala [123] have been shown to be under the influence of trauma. SES impacts reduced connectivity in neural networks involved in memory and emotion regulation [124]. In a recent study by Brody et al., data of 119 African American youths living in the rural South were used. The study measured poverty status and supportive parenting at ages 11–13 and 16–18. The study conducted brain imaging at age 25. This study applied resting-state fMRI to study two brain networks’ functional connectivity: (1) central-executive and (2) emotion-regulation. The authors found that more years spent in poverty was associated with lower levels of connectivity in both neural networks; however, this was more robust among young adults who received low levels of supportive parenting. The study did not show an effect of income on connectivity in the presence of high levels of positive and supportive parenting [124]. In a study that analyzed data from a prospective longitudinal study of emotion development showed that lower income-to-needs ratio at preschool age was associated with reduced connectivity between hippocampus and amygdala and several regions at school age, including the cortex, lingual gyrus, posterior cingulate, and putamen. This study included preschoolers 3–5 years of age selected from the St. Louis area. Participants were followed for up to 12 years. Individuals underwent annual behavioral assessments. Participants also underwent neuroimaging at school age to measure brain resting-state functional connectivity with the left and right hippocampus and amygdala. This study showed that a lower income-to-needs ratio predicted a greater connectivity between the left hippocampus and the right superior frontal cortex and between the right amygdala and the right lingual gyrus. As this study showed, brain functional connectivity mediated the relationship between SES and depression [125]. Thus, low SES predicts reduced connectivity between the amygdala and hippocampus with brain regions, including the lingual gyrus, superior frontal cortex, posterior cingulate, as well as putamen [125]. Social adversities have cumulative (additive) effects on brain structures and functions that govern emotion regulation [85] and memory [126]; however, these effects may differ across demographic groups [60]. In part, these are due to parents’ health [127] and behaviors [128].

While our first finding documented a link between SES and working memory, our second suggested that this effect differed across demographic groups. Most of the literature on the effects of race, parental education, and income on memory have focused on additive rather than multiplicative effects of SES indicators and race. Our second finding can also be seen as a reflection of the MDRs. Many studies have shown more significant effects of SES on outcomes for White than Black American children [49,62,129]. For example, family SES has shown larger effects on ADHD [79], anxiety [71], aggression [45], tobacco dependence [45], school bonding [130], school performance [73,131], obesity [69], and health [68] for White than Black American children.

As a result of our second finding, parental education shows a more salient role in shaping White’s impulsivity than Black American children [67]. As a result of this pattern, higher than expected risk of poor self-rated health, obesity, poor mental health, chronic disease, impulsivity, aggression, smoking, and low school performance are observed in high SES Black American children [69,77,79]. These patterns are also called MDRs and seem robust as they hold across SES indicators, outcomes, population groups, birth cohorts, age groups, and settings [36,37]. The findings observed in this analysis, however, did not support MDRs.

As shown by this study and previous works [36], family SES differently influences Black and White children’s outcomes [76,132], children [54], adults [133], and older adults [134,135]. A society is equal only if parental education [45], educational attainment [74,78,136], employment [137], marital status [63], and coping [138,139] generate equal outcomes for Blacks and Whites. Parental education seems to generate unequal effects for Blacks and Whites, a pattern that indicates inequality due to social stratification, segregation, and racism [140,141,142,143,144,145,146,147,148,149].

MDRs, differential effects of parental education across racial groups, maybe due to racial discrimination in high SES Black families. Racial and ethnic discrimination affect the amygdala’s structure and function [150,151,152,153,154,155]. In a study in the US, 74 adults (43% women; 72% African American; 23% Hispanic; 32% homosexual/bisexual) reported their discrimination experience. The study also measured spontaneous amygdala activity and functional connectivity between the amygdala and other brain regions during resting-state functional magnetic resonance imaging (fMRI). In this study, greater experience of discrimination was associated with an increased level of spontaneous amygdala activity. Similarly, an increase in discrimination was associated with stronger functional connectivity between the amygdala and several neural regions such as the anterior insula, putamen, caudate, anterior cingulate, medial frontal gyrus. The most robust effect of discrimination was seen for the connectivity between the amygdala and thalamus [156]. As high SES, particularly high subjective SES, is a proxy of high not low discrimination [50,51,52,53,55,56,57,58,137], high SES Black American children still report lower than expected brain and behavior outcomes because of the effect of discrimination.

Differential effects of family SES indicators for Black and White families contribute to the transgenerational transmission of inequalities [45,67,68,69,70]. Differential effects of SES mean the same level of SES may generate unequal outcomes for the next generation, which results in the reproduction of inequalities across generations. However, most of the previous studies on MDRs have relied on self-reported outcomes. Thus, the evidence lacked biological studies that test the differential effects of SES on children’s brain imaging. This paper documented complex, non-linear, multiplicative effects of SES, and race on working memory.

The observed MDRs suggest that Black American children suffer from three jeopardies. The first risk is that they live in low-SES families. The second risk is that they have worse outcomes (working memory in this study). The third jeopardy is their SES shows a weaker impact on their brain development. The weakened effect of SES for Black children suggests that it is very difficult to improve the health outcomes and close the Black-White gaps. Policymakers should not expect drastic effects as a result of their interventions. These diminishing returns are likely to be due to unique stressors in Black people’s lives across all SES levels.

It should be emphasized that we see race as a social factor (as a proxy of social status, treatment by society, access to the opportunity structure, interpersonal discrimination, environmental injustices, societal obstacles, and historical injustice) on how people are treated by society. As our results suggested, race alters the implications of family SES for working memory not because Blacks are inferior or different than Whites, but because society has historically oppressed them and continues to discriminate against them. All these injustices take a toll in terms of health and development.

Our paper was on complex and multiplicative effects of social determinants on 9- and 10-year-old American children. Working memory is closely associated with executive functioning and is mainly performed in higher cortical areas, especially the prefrontal cortex (PFC) [157,158,159]. It is recognized that the PFC is one of the last regions of the brain to mature [157]. During preadolescence, the increase in gray matter volume is observed, especially in the PFC, among other frontal lobe regions [157]. Furthermore, it is recognized that females’ brains develop about, on average two years earlier than male brains, so they are more likely to have a late developing male brain than females [157].

### Limitations

All studies have some methodological and conceptual limitations. This study, which was a secondary analysis of existing data, is not an exception to this rule. Our first limitation was a cross-sectional analysis. As a result, we can only conclude associations, not causal effects. Our second limitation was a lack of inclusion of many confounders such as psychopathologies, learning disabilities, or physical health. Third, all our SES measures were reported by parents. Some measurement bias should be expected in the measurement of SES in this study. Also, the sample was not balanced regarding race and SES. Racial groups were also not comparable in their SES. Finally, brain development is behind in male than female children. This study, however, did not explore race by sex differences in social determinants of working memory. Built on intersectionality, future research may explore how groups based on the intersection of race, sex, SES, and place differ in the effects of SDoH on memory function.

## 5. Conclusions

Among American children, high parental education and household income correlate with better working memory. However, the effect of parental education is unequal across racial groups, with the marginal return of parental education being smaller for Black families than White people. Income, however, generates a similar outcome for Black and White families. Finally, parental education generates less outcome than the differential income of highly educated Black and White families. These findings have policy solutions for achieving equality. First, the solution to racial gaps lies beyond closing the SES inequalities. We should address barriers that interfere with the SES from generating equal outcomes for Blacks. Equalizing income may be a more effective way of equalizing outcomes than equalizing education, because more processes can interfere with the return of education than income. More research is needed on how we can equalize Black and White families for the effect of SES on brain development. Social determinants’ influence on children’s brain development is complicated and multiplicative rather than simple and additive. Moderated mediation and mediated moderation models are more realistic than simple additive models.

## Figures and Tables

**Table 1:** Presents the descriptive statistics of the pooled sample and by race.

Level	All	White	Black	Asian	Other/Mixed	*p*
	*n* = 10,414	*n* = 6897	*n* = 1515	*n* = 234	*n* = 1768	
	Mean (SD)	Mean (SD)	Mean (SD)	Mean (SD)	Mean (SD)	
Age (Months)	118.96 (7.46)	119.03 (7.49)	118.89 (7.23)	119.40 (7.77)	118.65 (7.51)	0.187
Card Sorting Score	97.10 (15.26)	98.29 (15.07)	91.31 (13.98)	102.36 (17.94)	96.73 (15.46)	<0.001
	n (%)	n (%)	n (%)	n (%)	n (%)	
Parental education						
<HS Diploma	385 (3.7)	145 (2.1)	123 (8.1)	6 (2.6)	111 (6.3)	<0.001
HS Diploma/GED	862 (8.3)	327 (4.7)	340 (22.4)	3 (1.3)	192 (10.9)	
Some College	2674 (25.7)	1462 (21.2)	600 (39.6)	18 (7.7)	594 (33.6)	
Bachelor	2766 (26.6)	2057 (29.8)	230 (15.2)	65 (27.8)	414 (23.4)	
Post Graduate Degree	3727 (35.8)	2906 (42.1)	222 (14.7)	142 (60.7)	457 (25.8)	
Household Income						
<50 K	2997 (28.8)	1259 (18.3)	999 (65.9)	36 (15.4)	703 (39.8)	<0.001
>= 50 K & <100 K	2974 (28.6)	2104 (30.5)	335 (22.1)	54 (23.1)	481 (27.2)	
>= 100 K	4443 (42.7)	3534 (51.2)	181 (11.9)	144 (61.5)	584 (33.0)	
Latino						
No	8451 (81.2)	5737 (83.2)	1439 (95.0)	215 (91.9)	1060 (60.0)	<0.001
Yes	1963 (18.8)	1160 (16.8)	76 (5.0)	19 (8.1)	708 (40.0)	
Sex						
Female	4996 (48.0)	3254 (47.2)	760 (50.2)	117 (50.0)	865 (48.9)	0.128
Male	5418 (52.0)	3643 (52.8)	755 (49.8)	117 (50.0)	903 (51.1)	
Married Family						
No	3165 (30.4)	1415 (20.5)	1058 (69.8)	33 (14.1)	659 (37.3)	<0.001
Yes	7249 (69.6)	5482 (79.5)	457 (30.2)	201 (85.9)	1109 (62.7)	

**Table 2:** Effect sizes and % variance explained.

	*Model 1*	*Model 2*	*Model 3*	*Model 4*	*Model 5*	*Model 6*
n	11,315	10,418	10,414	10,418	11,315	10,414
R-squared	0.0454	0.03947	0.04545	0.03963	0.04696	0.04681
ΔR-squared	0.01244	0.00804	0.0062	0.02256	0.02922	0.01717
% Variance	1.24%	0.8%	0.62%	2.26%	2.92%	1.72%

**Table 3:** Mixed-effects regressions in the pooled sample (*n* = 10418).

Characteristics	b	SE	*p*	Sig
*Model 1*				
Parental Education (HS Diploma)	1.58	0.83	0.056	#
Parental Education (Some College)	2.86	0.75	<0.001	***
Parental Education (Bachelor)	4.84	0.78	<0.001	***
Parental Education (Graduate Degree)	6.69	0.77	<0.001	***
*Model 2*				
Household Income (50–100 K)	3.02	0.44	<0.001	***
Household Income (100 + K)	4.20	0.46	<0.001	***
*Model 3*				
Parental Education (HS Diploma)	0.95	0.94	0.311	
Parental Education (Some College)	2.04	0.85	0.017	*
Parental Education (Bachelor)	3.46	0.91	<0.001	***
Parental Education (Graduate Degree)	5.18	0.92	<0.001	***
Household Income (50–100 K)	2.21	0.52	<0.001	***
Household Income (100 + K)	1.88	0.47	<0.001	***
*Model 4*				
Household Income (50–100 K)	3.92	0.56	<0.001	***
Household Income (100 + K)	2.82	0.57	<0.001	***
Race (Black)	−4.96	0.69	<0.001	***
Race (Asian)	3.76	2.55	0.140	
Race (Other/Mixed)	−0.77	0.72	0.284	
Household Income (50–100 K) × Black	0.26	1.11	0.818	
Household Income (100 + K) × Black	0.66	1.34	0.624	
Household Income (50–100 K) × Asian	−2.18	3.27	0.505	
Household Income (100 + K) × Asian	−0.08	2.84	0.977	
Household Income (50–100 K) × Other/Mix	0.66	1.05	0.527	
Household Income (100 + K) × Other/Mix	0.86	0.99	0.385	
*Model 5*				
Parental Education (HS Diploma)	3.49	1.31	0.008	**
Parental Education (Some College)	3.87	1.14	0.001	***
Parental Education (Bachelor)	6.30	1.15	<0.001	***
Parental Education (Graduate Degree)	7.55	1.14	<0.001	***
Race (Black)	−1.77	1.66	0.286	
Race (Asian)	1.86	5.64	0.742	
Race (Other/Mixed)	0.49	1.64	0.768	
Parental Education (HS Diploma) × Black	−4.45	1.94	0.022	*
Parental Education (Some College) × Black	−2.44	1.77	0.169	
Parental Education (Bachelor) × Black	−3.62	1.93	0.060	#
Parental Education (Graduate Degree) × Black	−2.92	1.94	0.132	
Parental Education (HS Diploma) × Asian	−11.19	9.30	0.229	
Parental Education (Some College) × Asian	0.62	6.63	0.926	
Parental Education (Bachelor) × Asian	−1.26	5.90	0.831	
Parental Education (Graduate Degree) × Asian	1.83	5.76	0.751	
Parental Education (HS Diploma) × Other/Mix	−1.34	2.07	0.517	
Parental Education (Some College) × Other/Mix	−0.81	1.79	0.650	
Parental Education (Bachelor) × Other/Mix	−2.32	1.82	0.203	
Parental Education (Graduate Degree) × Other/Mix	0.80	1.80	0.659	
*Model 6*				
Parental Education (HS Diploma)	2.58	1.51	0.089	#
Parental Education (Some College)	2.80	1.35	0.038	*
Parental Education (Bachelor)	4.71	1.37	0.001	***
Parental Education (Graduate Degree)	5.86	1.37	<0.001	***
Household Income (50–100 K)	1.85	0.47	<0.001	***
Household Income (100 + K)	2.14	0.52	<0.001	***
Race (Black)	−2.34	1.89	0.216	
Race (Asian)	2.09	6.16	0.735	
Race (Other/Mixed)	0.80	1.89	0.673	
Parental Education (HS Diploma) × Black	−3.60	2.20	0.101	
Parental Education (Some College) × Black	−1.23	2.01	0.542	
Parental Education (Bachelor) × Black	−2.74	2.16	0.203	
Parental Education (Graduate Degree) × Black	−1.86	2.16	0.389	
Parental Education (HS Diploma) × Asian	−10.60	10.58	0.316	
Parental Education (Some College) × Asian	0.27	7.09	0.969	
Parental Education (Bachelor) × Asian	−1.36	6.44	0.833	
Parental Education (Graduate Degree) × Asian	2.11	6.29	0.738	
Parental Education (HS Diploma) × Other/Mix	−1.16	2.33	0.620	
Parental Education (Some College) × Other/Mix	−1.00	2.03	0.621	
Parental Education (Bachelor) × Other/Mix	−2.36	2.06	0.252	
Parental Education (Graduate Degree) × Other/Mix	0.52	2.04	0.800	

# *p* < 0.1, * *p* < 0.05, ** *p* < 0.01, *** *p* < 0.001.
